# Assessing the Function of Porcine A Kinase-Interacting Protein 1 (AKIP1) In Vitro—A Central Regulator of Oxidative Stress and Mitochondrial Functions

**DOI:** 10.3390/ijms26167759

**Published:** 2025-08-11

**Authors:** Agnieszka Bak, Arne Hinrichs, Anna Schwaiger, Tobias Fromme, Andrea Fischer, Mayuko Kurome, Valeri Zakhartchenko, Barbara Kessler, Martin Klingenspor, Eckhard Wolf, Angelika Schnieke, Konrad Fischer

**Affiliations:** 1 Chair of Livestock Biotechnology, School of Life Sciences Weihenstephan, Technische Universität München, 85354 Freising, Germany; 2 Chair of Molecular Animal Breeding and Biotechnology, Ludwig-Maximillians-Universität München, 85764 Oberschleissheim, Germany; 3 Chair of Molecular Nutritional Medicine, School of Life Sciences Weihenstephan, Technische Universität München, 85354 Freising, Germany

**Keywords:** A Kinase-Interacting Protein 1, AKIP1, oxidative stress, mitochondria, ROS production, pig, organ transplantation, ischemia–reperfusion injury

## Abstract

Oxidative stress plays a central role in numerous conditions, including cancer, cardiovascular and neurodegenerative diseases, diabetes, chronic inflammation, and organ transplantation. In transplantation, oxidative stress leads to mitochondrial dysfunction, DNA and protein damage, lipid peroxidation, and activation of pro-inflammatory pathways such as NF-κB, ultimately impairing cell viability and organ function. A Kinase-Interacting Protein 1 (AKIP1) has been linked to oxidative stress regulation in transgenic mouse models. To investigate this further in a livestock setting, we generated AKIP1 transgenic pigs and assessed AKIP1’s protective role against oxidative-stress-induced cell death, including apoptosis, necrosis, and ferroptosis in vitro. Our cellular analyses revealed reduced apoptosis (caspase-3/7 activity), suppressed MPTP-mediated necrosis, and decreased lipid peroxidation, suggesting protection from ferroptosis. Additionally, we observed lower mitochondrial superoxide production and enhanced mitochondrial respiration and recovery following H_2_O_2_-induced oxidative challenge. This is the first study to examine AKIP1 in porcine cells, providing a unique and translational platform for studying oxidative injury in a physiologically relevant species. Our in vitro data reveal that AKIP1 overexpression enhances antioxidant defenses and mitochondrial stability, offering future potential for improving graft survival in xenotransplantation.

## 1. Introduction

Oxidative stress plays a pivotal role in initiating various cell death pathways, including apoptosis, necrosis, and ferroptosis [1]. These processes are driven by the excessive accumulation of reactive oxygen species (ROS) and reactive nitrogen species (RNS), which overwhelm the cell’s antioxidant defenses and disrupt cellular homeostasis [2]. Mitochondria are particularly vulnerable to oxidative stress, as they are both a major source and target of ROS [3]. The impairment of mitochondrial function under these conditions can lead to further ROS production, creating a vicious cycle that exacerbates cellular damage and activates multiple cell death pathways. Central to this process is the disruption of mitochondrial respiratory functions, which normally help to maintain cellular energy homeostasis, calcium balance, and ROS detoxification. When oxidative stress impairs the electron transport chain, it results in electron leakage and the formation of superoxide (O_2−_), which is subsequently converted into harmful intermediates such as hydrogen peroxide (H_2_O_2_) and hydroxyl radicals (OH•) [4,5,6,7]. Although mitochondria possess intrinsic antioxidant mechanisms, including superoxide dismutase (SOD) and glutathione peroxidase, excessive ROS can overwhelm these defenses, leading to mitochondrial dysfunction, loss of membrane integrity, and the initiation of cell death pathways like apoptosis and ferroptosis [8,9].

Ischemia–reperfusion (I/R) injury exemplifies a pathological condition where oxidative stress and mitochondrial damage are critically involved. I/R injury occurs during the restoration of blood flow following a period of ischemia, a state of reduced blood and oxygen supply to tissues. This condition is prevalent in clinical scenarios such as stroke, myocardial infarction, and organ transplantation [10,11]. During the ischemic phase, the lack of oxygen impairs ATP synthesis, leading to a switch to anaerobic metabolism and an accumulation of metabolic byproducts [12,13]. Upon reperfusion, the sudden influx of oxygen into the previously ischemic tissue leads to a burst of ROS production as the overwhelmed mitochondrial respiratory chain releases excessive ROS, further triggering cellular damage. The resulting oxidative damage provokes various cell death pathways simultaneously, complicating therapeutic interventions aimed at reducing graft injury and improving recovery in organ transplantation. Key enzymes such as xanthine oxidase, NADPH oxidase, and uncoupled nitric oxide synthase, along with mitochondrial sources of ROS, have been identified as potential therapeutic targets to mitigate reperfusion-induced damage [4,9].

Among the cell death pathways activated during I/R injury, ferroptosis has gained attention for its unique association with mitochondrial dysfunction and oxidative stress. Unlike apoptosis and necrosis, ferroptosis is characterized by iron-dependent lipid peroxidation and significant mitochondrial alterations, including diminished cristae density, increased membrane permeability, and structural disintegration [7,14,15]. During the reperfusion phase, the heightened mitochondrial activity and reduced glutathione peroxidase 4 (GPX4) activity lead to an accumulation of lipid peroxides, triggering ferroptosis and exacerbating mitochondrial damage [16,17]. Understanding the complex interplay between oxidative stress, mitochondrial dysfunction, and cell death pathways is crucial for developing effective therapies to mitigate I/R injury [18]. Strategies aimed at reducing ROS production, enhancing antioxidant defenses, and stabilizing mitochondrial function may offer promising approaches to limit reperfusion-induced organ damage and improve outcomes in conditions such as organ transplantation. This highlights the need for continued research into the molecular mechanisms of oxidative stress and cell death, as well as the identification of genes upregulated by oxidative stress.

**A Kinase-Interacting Protein 1:** Initially identified as breast cancer-associated gene 3 (BCA3), A Kinase-Interacting Protein 1 (AKIP1) exhibits upregulation in various cancer types and is also present in most normal tissues. In cancer cells, AKIP1 influences NF-κB and Protein Kinase A (PKA) activity. Its diverse cellular localization, spanning the cytoplasm, nucleus, and mitochondria, suggests multiple cellular functions [19,20,21]. Additionally, AKIP1 serves as an early-response protein triggered by oxidative stress [21]. Experiments manipulating AKIP1 expression in cultured cardiomyocytes have revealed its promotion of mitochondrial respiration while mitigating mitochondrial ROS emissions, resulting in diminished I/R injury. Surprisingly, AKIP1′s protective role in I/R injury cannot be solely attributed to its interactions with signaling molecules like NF-κB, PKA, or AKT, as observed in cancer [20]. Instead, AKIP1 exhibits a high concentration in cardiac mitochondria and prevents calcium-induced mitochondrial swelling, indicative of reduced mitochondrial permeability transition pore formation [20]. Additionally, AKIP1 serves as a responsive regulator to oxidative stress, as evidenced by the protective effect observed in AKIP1 transgenic mice against ischemic injury, attributed to improved mitochondrial integrity and MPTP stabilization. Our aim was to create AKIP1 transgenic pigs to enable the investigation of AKIP1 overexpression in a large animal model. We preliminarily evaluated its effects on H_2_O_2_-induced oxidative cell damage and cell death pathways in vitro.

## 2. Results

### 2.1. Generation of the Porcine AKIP1 Model

The novel AKIP1 transgenic pig line (AKIP1-TG) was generated by transfection of wild-type kidney fibroblasts with a porcine AKIP1 transgene under the control of a cytokine-inducible CAG promoter, a selection of high-expressing cells, and subsequent somatic cell nuclear transfer to generate a male founder pig. This animal was subsequently bred with wild-type pigs to generate the F1 generation. Cells and tissues from the founder animal, as well as from male and female pigs of the F1 generation, were isolated and used for subsequent measurements of cellular responses regulating oxidative damage and mitochondrial characteristics. AKIP1 transgenic pigs were housed for up to 2.5 years and were bred up to the F2 generation without any discernible adverse effects on animal health, anatomy, physiology, or breeding characteristics.

**Figure 1:**
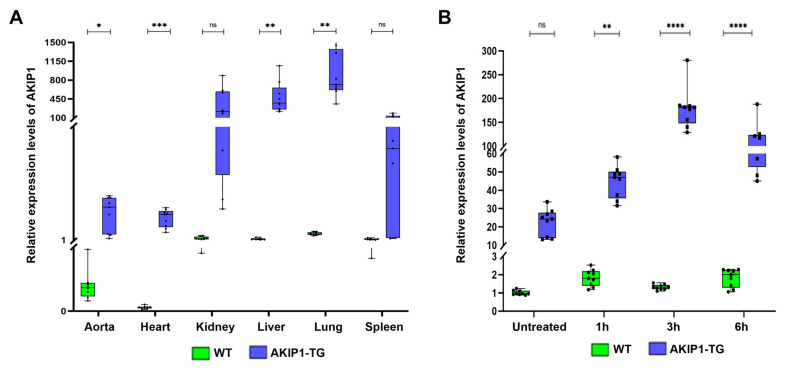
(**A**) Evaluation of AKIP1 expression levels in various tissues of wild-type (WT) and AKIP1 transgenic (AKIP1-TG) pigs, showing 8- to more than 400-fold increased AKIP1 expression levels. In total, 2 AKIP1-TG (1 animal of the F1 and 1 animal of the F2 generation) and 2 WT animals were used for data generation, with 3 technical replicates each. (**B**) Upregulation of AKIP1 expression in kidney fibroblasts after H_2_O_2_-induced oxidative stress. The cytokine-inducible CAG promoter increased AKIP1 expression more than 7-fold due to the addition of NF-κB binding sites after induction of oxidative stress. All values normalized to GAPDH expression levels.

Despite the random integration of only a single transgene copy, significantly elevated expression levels, 8- to over 400-fold increased compared to wild-type pigs, were observed in various organs, including the heart, aorta, liver, kidneys, spleen, and lungs (Figure 1A). To verify the effects of the cytokine-inducible CAG promoter, which is upregulated through NF-κB signaling due to the addition of 5 NF-κB binding sites [22], we assessed AKIP1 expression levels following oxidative stress, a known trigger of NF-κB pathway activation. H_2_O_2_-induced oxidative stress significantly enhanced AKIP1 expression, resulting in more than a 7-fold increase compared to untreated control cells (Figure 1B).

### 2.2. AKIP1 Overexpression Inhibits Cellular Death Pathways

**Figure 2:**
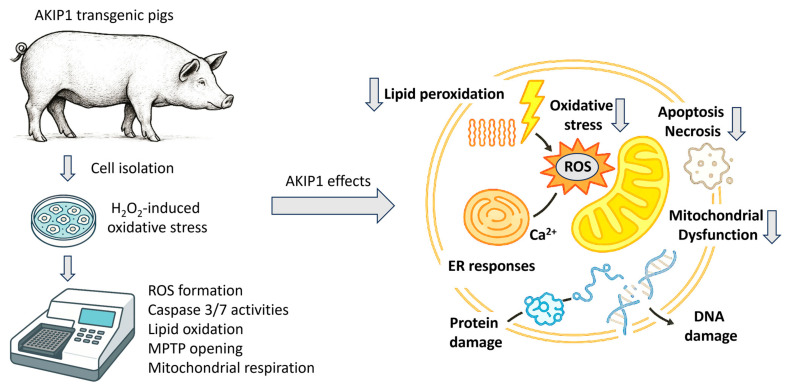
Experimental overview of in vitro characterizations of cells derived from AKIP1 transgenic pigs. Isolated cells were analyzed for various oxidative-stress-induced cell death pathways. In summary, AKIP1 expression led to reduced oxidative stress and decreased ROS production. Consequently, markers of apoptosis, necrosis, and lipid peroxidation were downregulated. Additionally, AKIP1-transgenic cells showed enhanced mitochondrial membrane stability and improved respiratory functions.

Oxidative damage is closely linked to apoptosis and other cell death pathways, as excessive ROS can damage DNA, proteins, and mitochondria, leading to the activation of apoptotic signaling pathways. We isolated kidney fibroblasts from AKIP1-TG pigs and used them for subsequent experiments (Figure 2).

**Figure 3:**
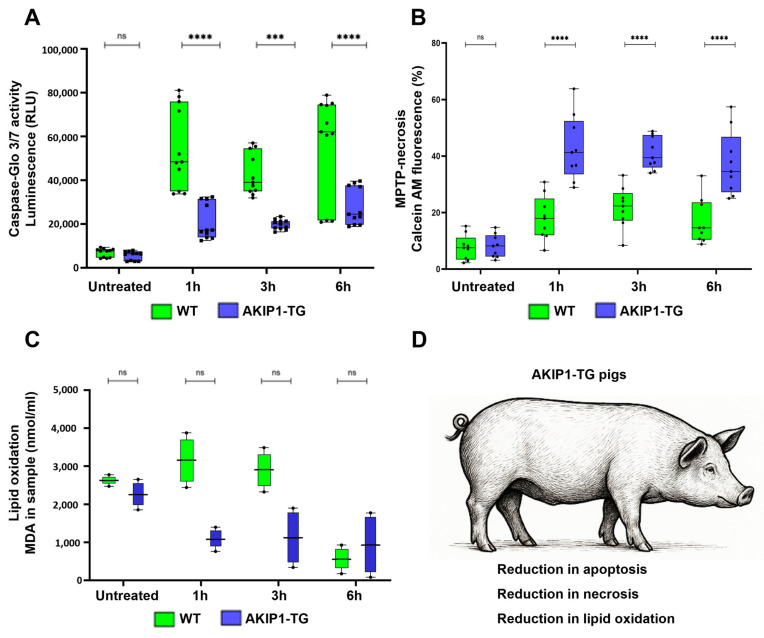
(**A**) Caspase-Glo 3/7 assay to quantify apoptosis due to caspase 3/7 activities after 1 h of oxidative stress with 100 µM of H_2_O_2_ and subsequent readout for up to 6 h. AKIP1 transgenic cells exhibited markedly reduced levels of apoptosis. (**B**) Implementation of the MitoProbe–Transition-pore assay to assess MPTP-mediated necrosis for up to 6 h after oxidative stress. Presented is the calcein fluorescence subsequent to CoCl_2_ addition. AKIP1 transgenic cells displayed heightened calcein fluorescence, indicative of preserved outer mitochondrial membrane integrity and reduced incidence of MPTP-mediated necrosis. (**C**) Malondialdehyde (MDA) quantification after oxidative stress. MDA is a primary byproduct of lipid peroxidation. AKIP1 transgenic cells showed reduced MDA formation, representing reduced lipid peroxidation. (**D**) Summary of the effects of the AKIP1 transgene, reducing apoptotic cell death responses, MPTP-necrosis, and lipid oxidation. Cells from 2 AKIP1-TG and 2 WT animals were used for data generation.

To detect effects of AKIP1 on apoptosis, cells were incubated for 1 h with 100 µM of H_2_O_2_, and the activities of caspase-3 and caspase-7, the key enzymes involved in apoptosis, were subsequently measured for up to 6 h. For the AKIP1 transgenic cells (AKIP1-TG), a significant downregulation of apoptosis over all measured time points was found (Figure 3A). Furthermore, oxidative damage is linked to necrosis, as excessive ROS can cause severe mitochondrial dysfunction, ATP depletion, and loss of plasma membrane integrity, leading to unregulated cell death. Unlike apoptosis, necrosis induced by oxidative stress often triggers inflammation, as cellular contents are released into the extracellular space.

In our MPTP-mediated necrosis assay, we observed a stronger decrease in calcein fluorescence lasting up to 6 h after oxidative stress in wild-type cells, in contrast to AKIP1-TG cells. This finding suggests a significantly diminished occurrence of MPTP-mediated necrosis in AKIP1-TG cells (Figure 3B). Moreover, oxidative damage is a key driver of ferroptosis, a form of regulated cell death characterized by iron-dependent lipid peroxidation. Excessive ROS and disruptions in antioxidant defenses, such as glutathione depletion or GPX4 inactivation, lead to the accumulation of peroxidized lipids in cellular membranes. Initially, we directly measured malondialdehyde (MDA), a primary byproduct of lipid peroxidation, in wild-type and AKIP1-TG cells following oxidative stress. Although AKIP1-TG cells showed a clear trend toward reduced MDA formation, the results were not statistically significant (Figure 3C). All the main findings regarding the reduction in cell death pathways are summarized in Figure 3D. We subsequently examined the effects of AKIP1 on the expression levels of some of the key enzymes of the ferroptotic pathways, GPX4 and ACSL4 (Supplementary Figure S1A,B). Both enzymes were modulated in wild-type cells in response to oxidative stress. In AKIP1-TG cells, neither GPX4 nor ACSL4 showed significant changes. As MDA formation was reduced in AKIP1-TG cells, these results suggest that AKIP1 may mitigate ferroptosis through mechanisms independent of these pathways.

### 2.3. AKIP1 Expression Protects Mitochondria Against Oxidative Stress

**Figure 4:**
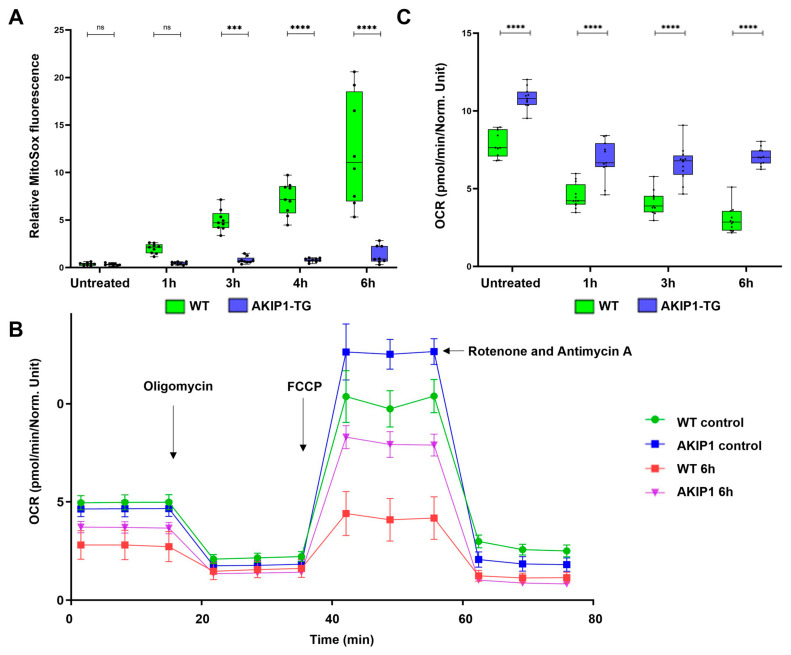
(**A**) Mitochondrial superoxide production subsequent to oxidative stress, induced by 100 µM of H_2_O_2_ for 1 h. AKIP1 transgenic cells demonstrated efficient suppression of superoxide production over the subsequent 6 h time period. (**B**) Seahorse assay demonstrating the oxygen consumption rate (OCR) of wild-type and AKIP1 transgenic cells, without control, and up to 6 h after the oxidative stress. AKIP1 transgenic cells showed a higher maximal uncoupled OCR compared to wild-type cells, representing higher mitochondrial activity and faster mitochondrial recovery. Oligomycin was used to measure the ATP-linked OCR. To determine maximal respiration, the uncoupler FCCP was used. Rotenone and Antimycin A were used to determine the non-mitochondrial respiration. (**C**) Increased maximal oxygen consumption rate (OCR) and maximal respiratory capacity of AKIP1-TG cells after oxidative stress. Cells from 1 AKIP1-TG animal and 1 WT animal were used for data generation.

To elucidate the impact of AKIP1 on mitochondrial functions, we evaluated mitochondrial superoxide production and the mitochondrial respiratory capacity under oxidative stress. When the electron transport chain becomes impaired or overloaded, electrons leak and react with oxygen, forming superoxide, a primary ROS. Excessive mitochondrial superoxide contributes to oxidative stress by overwhelming cellular antioxidant defenses. In wild-type cells, incubation for 1 h with H_2_O_2_ markedly increased superoxide production over the following 6 h time period. Conversely, AKIP1-TG cells exhibited almost no increase, despite the robust oxidative challenge (Figure 4A). Additionally, we assessed the mitochondrial respiratory functions and oxygen consumption rates of wild-type and AKIP1-TG cells following exposure to oxidative stress. The results of the Seahorse assay are shown in Figure 4B; the calculated maximal oxygen consumption rates are shown in Figure 4C. Independent experimental replications are shown in Supplementary Figure S2A,B. AKIP1-TG cells demonstrated an enhanced maximal respiratory capacity and accelerated mitochondrial recovery post-oxidative stress compared to wild-type cells. This reveals that AKIP1 plays a protective role in maintaining mitochondrial respiratory functions and mitigating oxidative damage.

## 3. Discussion

This study—using a novel AKIP1 transgenic pig model—highlights the protective role of AKIP1 against oxidative stress-induced cell death and mitochondrial dysfunctions. This study presents the first investigation of AKIP1 expression and function in a porcine model, marking a significant step forward in translational research on oxidative damage and potentially I/R injury in large animals. While our findings are based on in vitro data, this model provides a unique and highly relevant platform for studying cellular responses in a species with physiological and anatomical characteristics closer to humans than rodent models. A key limitation of this work currently lies in the lack of reliable antibodies for porcine AKIP1, which currently prevents robust protein-level validation through standard techniques. This reflects a broader challenge in porcine molecular biology, where the limited availability of species–specific reagents hampers downstream validation. Additionally, despite AKIP1′s known association with several signaling pathways in human and murine systems, detailed mechanistic insights remain elusive, even after decades of research. Our study focuses on establishing foundational expression data and functional responses in porcine fibroblasts, laying the groundwork for future mechanistic and translational investigations. Given the time-intensive development and breeding of this genetically modified porcine model, and its potential applicability to xenotransplantation and I/R studies, this work offers a unique new tool to the field. The high baseline expression of AKIP1 across all tested tissues, even in the absence of oxidative stress, could be explained by the fact that the 5xNF-κB promoter element used in our construct may allow for constitutive activation of the transgene, potentially through a positive feedback mechanism. AKIP1 is known to interact with the NF-κB signaling pathway and has been reported to enhance NF-κB transcriptional activity by promoting the nuclear translocation of the p65 subunit. Therefore, it is possible that once AKIP1 expression is initiated, it could further amplify its own transcription via NF-κB activation. Additionally, AKIP1 has been shown to localize in the nucleus and bind directly to the p65 subunit of NF-κB, serving as a coactivator that enhances NF-κB-dependent gene expression. This interaction could further support a feedback loop leading to sustained or elevated transgene expression in tissues with basal NF-κB activity.

Although we did not directly provide data on the relationship between oxidative stress and inflammation, the interplay between both, particularly via NF-κB signaling, is already well established. Experimental studies in transplantation have shown that oxidative injury activates NF-κB, leading to inflammatory cytokine release and graft dysfunction. Antioxidant treatment was shown to reduce ROS and NF-κB-mediated cytokine expression (IL-1β, TNF-α), improving islet transplant outcomes [23]. Similarly, oxidative stress in liver grafts triggered NF-κB-driven inflammation and remote lung injury, mitigated by NF-κB inhibition [24]. In cancer models, ROS was shown to promote tumor development and progression via NF-κB-dependent inflammatory signaling [25]. These findings underscore the importance of this axis in both contexts. Our findings for porcine AKIP1 could verify murine AKIP1 functions, modulating oxidative stress responses; enhance cellular survival; and improve mitochondrial functions [20,26]. We used a porcine AKIP1 genomic construct, as porcine AKIP1 transcripts have not yet been studied in detail, and the functionality of murine and human AKIP1 in porcine cells is unknown. The results obtained from the porcine transgene aligned with those from murine and human AKIP1 genes. The inhibition of apoptosis in porcine AKIP1-TG cells, as demonstrated by reduced caspase-3 and caspase-7 activity following oxidative stress, confirms findings from earlier studies of cardiomyocytes and cancer models [21,27,28]. These studies revealed that AKIP1 stabilizes anti-apoptotic proteins, such as Bcl-2, through interactions with PKA signaling. This protective mechanism likely underpins the observed suppression of apoptotic pathways in AKIP1-TG cells, emphasizing its role in cellular survival under oxidative stress conditions [26]. In addition to apoptosis, AKIP1 also mitigates necrosis in the porcine model, as evidenced by the reduced mitochondrial dysfunction; stabilizes mitochondrial membranes; and inhibits pathological MPTP opening.

By limiting unregulated cell death and associated inflammatory responses, AKIP1 demonstrates its potential to protect cells from the dual threats of apoptosis and necrosis under oxidative stress.

Interestingly, while ferroptosis is another oxidative-stress-driven cell death pathway, despite a strong reduction in lipid peroxidation in AKIP1-TG cells, no significant changes in the expression of the key ferroptotic enzymes, GPX4 or ACSL4, could be detected in AKIP1-TG cells. This result contrasts with the significant modulation of these enzymes in wild-type cells. While it was anticipated that AKIP1-TG cells might show distinct alterations, the findings suggest that AKIP1 may mitigate ferroptosis through mechanisms independent of canonical pathways. This hypothesis is supported by AKIP1′s known ability to enhance antioxidant defenses, indicating the need for further investigation into its indirect role in ferroptosis regulation. Future studies could explore non-canonical mechanisms, such as the maintenance of cellular glutathione levels, indirect lipid peroxidation suppression, or the modulation of ferroptosis inhibitors. Lipidomic analyses could also offer valuable insights into changes in lipid peroxidation profiles, shedding light on how AKIP1 influences ferroptotic processes. In this context, it would be very interesting to investigate the function of AKIP1 in the regulation of intracellular Ca^2+^ homeostasis. Mitochondria play a crucial role in buffering intracellular Ca^2+^, and their functional state directly affects Ca^2+^ homeostasis. In neonatal rat ventricular cardiomyocytes, as well as in our study, AKIP1 overexpression enhanced mitochondrial respiration without increasing superoxide production, revealing improved mitochondrial efficiency. This enhancement was independent of mitochondrial biogenesis or changes in membrane potential, indicating a direct effect on mitochondrial function [19]. By enhancing mitochondrial efficiency, AKIP1 may indirectly support the maintenance of intracellular Ca^2+^ levels, preventing Ca^2+^ overload that can lead to cell injury or death. Additionally, AKIP1 has been shown to interact with components of the mitochondrial permeability transition pore, such as ATP synthase. Overexpression of AKIP1 in cardiac-specific transgenic mice reduced calcium-induced mitochondrial swelling, indicative of decreased MPTP opening [20]. Since MPTP opening is a critical event leading to mitochondrial dysfunction and altered Ca^2+^ homeostasis, AKIP1′s stabilizing effect on the MPTP suggests a protective role in maintaining Ca^2+^ regulation within cells. Additionally, one of the targets of AKIP1, Protein Kinase A (PKA), also regulates intracellular Ca^2+^ homeostasis. PKA plays a central role in regulating intracellular Ca^2+^ dynamics by phosphorylating key proteins involved in calcium release and reuptake. PKA phosphorylates ryanodine receptors on the sarcoplasmic or endoplasmic reticulum, enhancing their sensitivity and promoting Ca^2+^ release into the cytosol [29,30].

It also phosphorylates phospholamban, relieving its inhibition on the sarco/endoplasmic reticulum Ca^2+^-ATPase (SERCA), which increases Ca^2+^ reuptake into storage organelles [31]. Moreover, PKA can modulate inositol 1,4,5-trisphosphate receptors (IP3Rs), further influencing Ca^2+^ release. These effects could contribute to explaining the observed effects of AKIP1 on lipid peroxidation and ferroptosis.

The impact of AKIP1 on mitochondrial function was particularly striking. AKIP1-TG cells demonstrated enhanced maximal respiratory capacity and accelerated recovery following oxidative stress, as revealed by Seahorse assay data. These results align with prior studies that reported AKIP1′s ability to stabilize mitochondrial dynamics, maintain oxidative phosphorylation, and boost ATP production [21,26]. Additionally, AKIP1-TG cells exhibited reduced mitochondrial superoxide production compared to wild-type cells, despite exposure to robust oxidative challenges. These observations underscore AKIP1′s critical role in preserving mitochondrial efficiency and mitigating oxidative damage, further strengthening its position as a key regulator of mitochondrial resilience. A deeper investigation into mitochondrial dynamics could provide clarity on how AKIP1 regulates mitochondrial fusion and fission proteins, such as MFN2 and DRP1, under oxidative stress [32,33]. Proteomic and transcriptomic profiling would also be valuable for identifying downstream targets of AKIP1 in apoptosis, necrosis, and ferroptosis pathways while uncovering interactions with other redox-sensitive transcription factors beyond NF-κB, such as Nrf2 [34]. The unique, distinctive feature is the future availability of a livestock model offering AKIP1 overexpression. Using this model and expanding the scope to in vivo experiments could provide additional insights into AKIP1′s protective role in whole-organ systems. Previous studies have reported AKIP1 upregulation in various cancers. However, based on our current understanding, we believe this upregulation likely reflects a protective response by cancer cells to oxidative stress and a mechanism to support their high energy demands rather than a direct oncogenic role of AKIP1 itself. Importantly, in our own experience, we have monitored AKIP1 transgenic pigs over multiple generations, with some individuals observed for more than three years. To date, we have not observed any signs of tumor development in these animals. On the contrary, all animals have remained healthy and shown normal development and behavior, which supports the safety of AKIP1 overexpression in this context. AKIP1-TG pigs could be utilized in models of ischemia–reperfusion injury or chronic oxidative stress to study long-term effects on organ function and survival. Investigating AKIP1′s role in aging and age-related diseases would also be of interest, as older AKIP1-TG pigs could provide a unique platform to assess its impact on longevity and age-associated oxidative damage. Testing pharmacological strategies to upregulate AKIP1 or deliver AKIP1 mimetics in disease models could offer promising avenues for treating oxidative-stress-related conditions, such as cardiovascular diseases, neurodegenerative disorders, and organ damage from ischemia–reperfusion injury. Incorporating the AKIP1 transgene into animals of our multi-modified lines for xenotransplantation [35,36] will explore new pathways of organ rejection and potentially enhance graft survival significantly.

## 4. Materials and Methods

**Animal welfare**

Animal experiments were approved by the Government of Upper Bavaria and performed according to the German Animal Welfare Act and European Union Normative for Care and Use of Experimental Animals.

**AKIP1 transgene construct**

The AKIP1 transgene construct consisted of an improved, cytokine-inducible 2 kb CAG promoter [22] (5 NfkB binding sites, CMV enhancer, chicken beta-actin promoter, rabbit beta-globin splice acceptor), a 6.5 kb genomic porcine AKIP1 sequence, and the bovine growth hormone (BGH) poly A sequence (Supplementary Figure S3). This construct ensures the expression of all annotated porcine *AKIP1* transcripts. Wild-type porcine kidney fibroblasts were transfected with the AKIP1 construct, selected for high-expressing clones, and subsequently used for somatic cell nuclear transfer.

**Somatic cell nuclear transfer**

Nuclear transfer was performed as described previously [35,37,38]. In short, donor cells were arrested at the GO/G1 phase by serum deprivation. Oocytes isolated from prepubertal gilts were matured in vitro and enucleated; single-donor cells were inserted into the perivitelline space; and then, cell fusion and oocyte activation were induced by an electric pulse. Reconstructed embryos were transferred into the oviducts of hormonally synchronized recipient gilts by mid-ventral laparotomy.

**Cell isolation and cell culture**

Primary porcine kidney fibroblasts were isolated from porcine kidney tissue. Tissue pieces were washed with PBS buffer (supplemented with 1x Pen/Strep), minced into approx. 1 mm^3^ pieces using a scalpel, and washed again 2–3 times until the supernatant remained clear. After removal of PBS, tissue pieces were resuspended in 10 mL of PBS with 0.1% collagenase A (Sigma Aldrich, Steinheim, Germany, cat. no. COLLA-RO) and incubated at 37 °C while stirring for 30 min. After incubation, flasks were filled up with 10 mL of DMEM and centrifuged at 300× *g* for 5 min. Cell pellets were resuspended in 5 mL of medium with antibiotics (DMEM, 20% FCS, 1 mmol/L sodium pyruvate, 1x NEAA, 2 mmol/L Ala/Glu, 1x Pen/Strep) and transferred into a T150 flask.

**RNA isolation and real-time PCR**

Total RNA was isolated from cells and tissues by using the Monarch Total RNA Miniprep Kit (New England Biolabs, Frankfurt am Main, Germany, cat. no. T2010S), and cDNA was synthesized based on the LunaScript RT Master Mix Kit (New England Biolabs, Germany, cat. no. E3025S) according to the manufacturer’s instructions. Relative gene expression levels were determined by quantitative real-time PCR (RT-PCR) using the QuantStudio 5 Real-Time PCR System (Thermo Fisher Scientific, Dreieich, Germany) and TaqMan Real-Time-PCR-Assays (Thermo Fisher Scientific, Germany, cat. no. 4352042), based on GAPDH as a reference gene. Primers and probes are listed in Supplementary Table S1.

**Oxidative stress induction**

Then, 24 h before performing stress tests, cells were split by using Accutase solution (Sigma-Aldrich, Germany), and 0.7 × 10^6^ cells were seeded in T25 bottles (4 times each for WT and AKIP1-TG). To cause oxidative stress [39], mimicking an ischemia–reperfusion injury, cells were washed 2 times with PBS and incubated for 1 h with 100 µM of H_2_O_2_, dissolved in standard DMEM medium without any supplements. Subsequently, cells were washed with PBS, and culture medium was added (DMEM, 20% FCS, 1 mmol/L sodium pyruvate, 1x NEAA, 2 mmol/L Ala/Glu) to mimic the reperfusion-like phase. Measurements were conducted after 1 h, 3 h, and 6 h by harvesting the cells using Accutase solution and performing individual assays.

**Caspase assay**

After stressing and harvesting cells as described before, caspase 3/7 activity [40] was measured with the Caspase-Glo 3/7 Assay System (Promega, Walldorf, Germany, cat. no. G8090) according to the manufacturer’s instructions. In total, 12,000 cells per well were used, and luminescence signals were measured immediately after gently mixing cells with Caspase-Glo 3/7 reagent in a FLUOstar Omega microplate reader with Voyager and MARS software (BMG Labtech, Ortenberg, Germany).

**Measurement of mitochondrial respiration**

To measure key parameters of mitochondrial function [41], the oxygen consumption rate (OCR) of cells during a mitochondrial stress test was detected by using a Seahorse XF Pro Analyzer (Agilent, Santa Clara, CA, USA). After H_2_O_2_ stressing and harvesting as described before, cells were seeded at a density of 30,000 cells/well on a Seahorse assay plate. Then, 1 h before measurements, the medium was replaced with XF DMEM medium; supplemented with 1 mM of pyruvate, 2 mM of glutamine, and 10 mM of glucose; and incubated for 1 h at 37 °C (without CO_2_). Three baseline OCR measurements were performed, followed by injection with oligomycin (5 µM) to measure the ATP-linked OCR. To determine maximal respiration, the uncoupler FCCP (1 µM) was used. Antimycin A (5 µM) and Rotenone (0.5 µM) were used to determine the non-mitochondrial respiration. In total, 3 independent measurements of 4 technical replicates each were performed for every experimental setting.

**Determination of mitochondrial ROS production**

ROS production [42] was determined by using the MitoSOX Mitochondrial Superoxide assay (Thermo Fisher Scientific, Dreieich, Germany, cat. no. M36008) according to the manufacturer’s instructions. After stressing and harvesting as described before, cells were washed with PBS, resuspended in HBSS (with calcium, magnesium, and no phenol red; Thermo Fisher Scientific, Dreieich, Germany) to a final volume of 0.5 mL, and incubated with MitoSOX (1 µM) for 20 min at 37 °C. Cells were centrifuged (300× *g*, 5 min), washed 2 times with HBSS, and resuspended to a final volume of 0.2 mL with HBSS. Fluorescence was measured using excitation/emission maxima of 510/580 nm with an Attune NxT Flow Cytometer (Thermo Fisher Scientific, Dreieich, Germany).

**Measuring mitochondrial permeability transition pore (MPTP) opening**

To directly measure the opening of the mitochondrial permeability transition pore [43], the dye calcein–acetoxymethyl ester was used. This dye is introduced into the cells, where it passively diffuses and accumulates within various cellular compartments, including the mitochondria. Once inside the cells, intracellular esterases enzymatically cleave the acetoxymethyl ester moiety of the calcein dye, liberating the highly polar fluorescent calcein molecule. However, due to its limited ability to pass mitochondrial or plasma membranes, calcein fluorescence was retained within the mitochondria for the duration of the experiment. The addition of CoCl_2_, which is impermeable to intact mitochondria, effectively quenches the fluorescence originating from cytosolic calcein, thereby limiting fluorescence to the mitochondria. MPTP opening facilitates the mixing of mitochondrial calcein with cytosolic CoCl_2_ and consequently leads to a decline in mitochondrial fluorescence. As a positive control for MPTP activation, cells loaded with calcein–acetoxymethyl ester and CoCl_2_ were subjected to ionomycin treatment, an ionophore facilitating the influx of excess Ca^2+^ into the cells, triggering pore opening and loss of mitochondrial calcein fluorescence.

MPTP opening was measured using the MitoProbe Transition Pore Assay (Thermo Fisher Scientific, Germany, cat. no. M34153) according to the manufacturer’s instructions. After stressing and harvesting as described before, cells were divided into 3 aliquots, and 2 μM of calcein AM was added to sample 1; 2 µM of calcein AM and 80 mM CoCl_2_ to sample 2; and 2 µM of calcein AM, 80 mM CoCl_2_, and 100 μM ionomycin to sample 3. After incubation at 37 °C for 15 min, cells were centrifuged (300× *g*, 5 min), washed 2 times with HBSS buffer, and resuspended in HBSS to a final volume of 0.2 mL. Fluorescence was measured using excitation/emission maxima at 488/517 nm with an Attune NxT Flow Cytometer (Thermo Fisher Scientific, Germany).

**Statistics**

Data are displayed as the mean +/− the standard deviation (SD) of the mean. Statistical significance was assessed using a threshold *p*-value of less than 0.05. For comparisons involving more than two groups with response variables such as relative expression or luminescence, a two-way ANOVA was conducted. Subsequently, post hoc Tukey’s and Sidak’s multiple comparisons tests were employed to detect differences within each group. Statistical analysis was conducted using the GraphPad Prism software (Version 8.0.1).

## Data Availability

The raw experimental data are available upon reasonable request from the corresponding author.

## Supplementary Materials

The following supporting information can be downloaded at https://www.mdpi.com/article/10.3390/ijms26167759/s1.
