# Supplementary material for: Assessing the Function of Porcine A Kinase-Interacting Protein 1 (AKIP1) In Vitro—A Central Regulator of Oxidative Stress and Mitochondrial Functions
Source: Int J Mol Sci. 2025 Aug 11;26(16):7759. doi: 10.3390/ijms26167759 (PMC12386592; doi:10.3390/ijms26167759)
Supplement: Supplementary file 1 [file ijms-26-07759-s001.zip › ijms-3772256-supplementary.pdf]

Supplementary tables

Table S1: Primer and probe sequences used for RT-PCR

Primer/Probe	Sequence (5′→3′)
ACSL4 forward	CGAAGGGAGTGATGATGCAT
ACSL4 revers	TGGTCAGAGAGTGTCAGTGG
ACSL4 probe	[FAM] CCTGGACTGGGACCAAAGGACA [BHQ1]
AKIP1 forward	TTCGGTGGAGCTTCCCAAAG
AKIP1 revers	CAGCTCCGACTTGCCTCTGT
AKIP1 probe	[FAM] ACTGTTCATCGCTGCCCGAG [BHQ1]
GPX4 forward	TGGTTTACGGATTCTGGCCT
GPX4 revers	CCTTGGGCTGGACTTTCATC
GPX4 probe	[FAM] CCAGTTTGGGAGGCAGGAGC [BHQ1]
poGAPDH forward	CGCGTGAACCATGAGAAGTATG
poGAPDH revers	GGTGCAGGAGGCATTGCT
poGAPDH probe	[FAM] AACTCCCTCAAGATCGT [BHQ1]

Supplementary figures

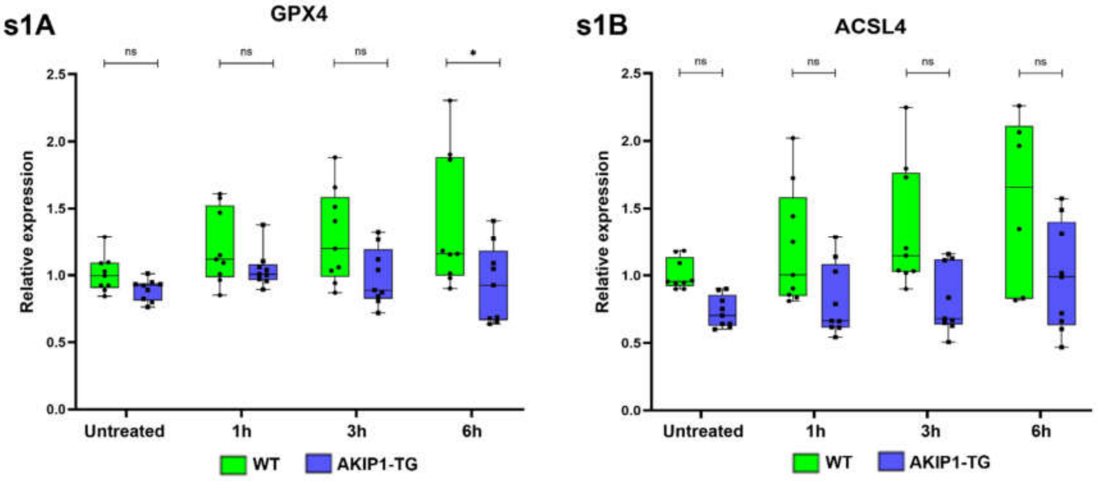

Figure S1: A) Anti-ferroptotic GPX4 and **B)** pro-ferroptotic ACSL4 expression pathways of wild-type (WT) and AKIP1 transgenic (AKIP1-TG) cells. WT cells showed a significantly increased expression of GPX4 after 6 hours and a slightly increased ACSL4 expression, compared to AKIP1-TG cells. All values normalized to GAPDH expression levels.

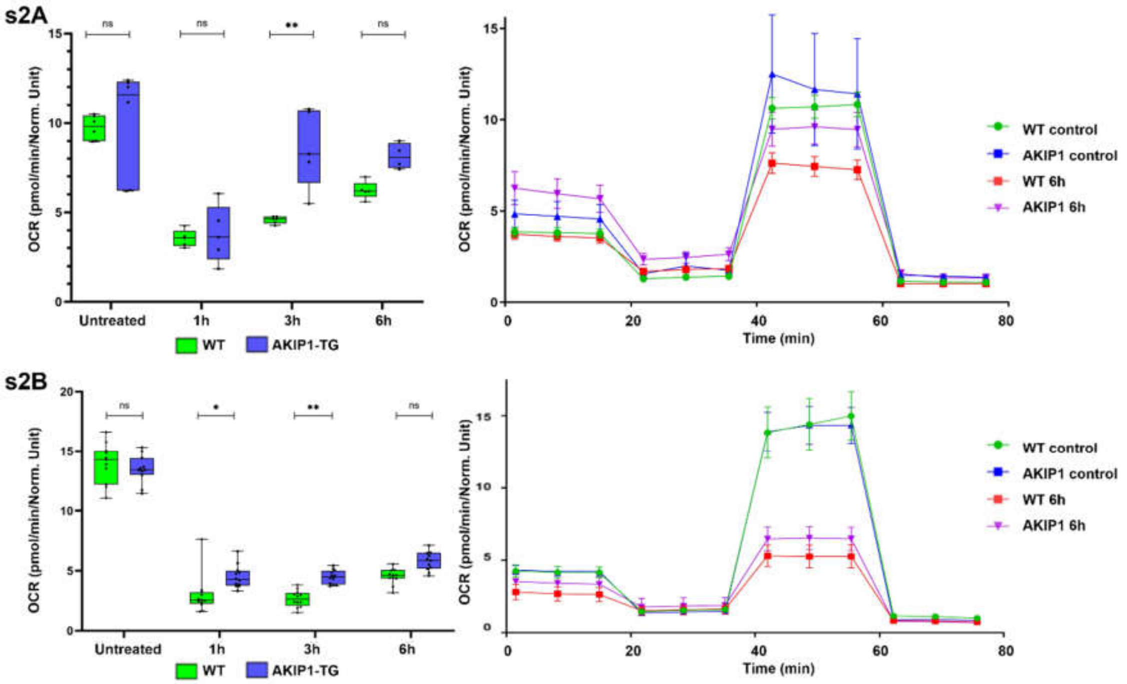

Figure S2: A) and B) Independent Seahorse assays demonstrating the oxygen consumption rate (OCR) of wild type and AKIP1 transgenic cells, without (control) and up to 6 hours after the oxidative stress. AKIP1 transgenic cells showed a higher maximal uncoupled OCR compared to wild type cells, representing higher mitochondrial activity and faster mitochondrial recovery.

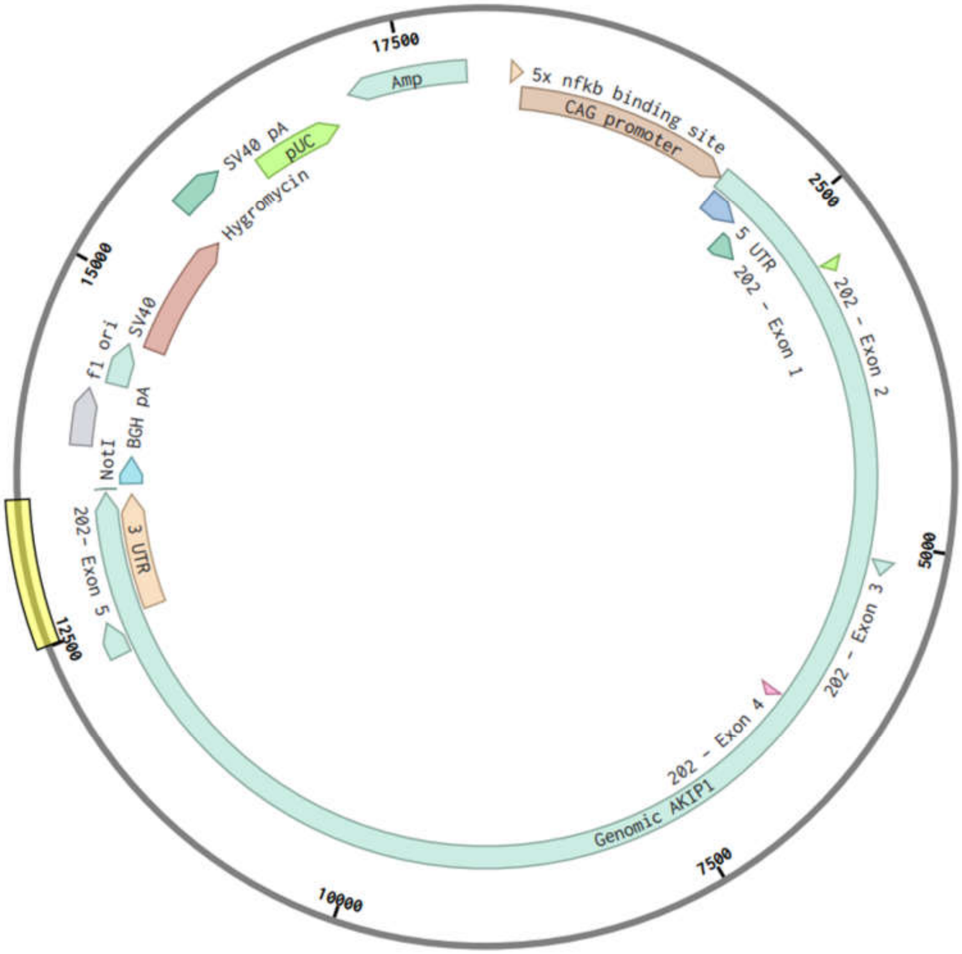

Figure S3: AKIP1 transgene construct based on a pcDNA3.1 hygro+ plasmid backbone, consisting of an improved, cytokine-inducible 2 kb CAG promoter (5x NfkB binding sites, CMV enhancer, chicken beta-actin promoter, rabbit beta-globin splice acceptor), a 6.5 kb genomic porcine AKIP1 sequence, and the bovine growth hormone (BGH) poly A sequence. Exons of transcript number 202 are annotated for better visualization of exon and intron structures.
